# Compliance with the consumption of iron and folate supplements by pregnant women in Mafikeng local municipality, North West province, South Africa

**DOI:** 10.4314/ahs.v17i3.8

**Published:** 2017-09

**Authors:** Xikombiso Mbhenyane, Matodzi Cherane

**Affiliations:** 1 Stellenbosch University, Human Nutrition; 2 University of Venda, Nutrition

**Keywords:** Iron and folate supplements, Mafikeng local municipality, North West province, South Africa

## Abstract

**Background:**

Anaemia due to iron deficiency is recognized as one of the major nutritional deficiencies in women and children in developing countries. Daily iron supplementation for pregnant women is recommended in many countries. The aim of the study was to investigate the factors that contribute to compliance to the consumption of iron and folate supplements by pregnant woman in Mafikeng local municipality, North West Province, South Africa.

**Research Methods:**

A mixed method of descriptive, exploratory and cross-sectional design was used. Ten clinics were used as a sample frame where 57 pregnant women and 10 health workers were purposefully and conveniently selected. Quantitative techniques were used to collect data on attendance, consumption and nutrition knowledge using the self-reported questionnaire by pregnant women, and structured interview for health workers. Qualitative design was used to conduct in - depth focus-group discussions to gather information on compliance to the consumption of supplements by pregnant women.

**Findings:**

The findings of the study revealed good antenatal clinic attendance, availability of supplements and 93% compliance to the consumption of iron and folate supplements.

**Recommendations:**

High compliance to the consumption of iron and folate supplements by pregnant women was reported, and this should be reinforced.

## Introduction and background and information

Anaemia in pregnancy is a major health problem in many developing countries where nutrient deficiency, malaria and other parasites infections contribute to increased maternal and pre-natal mortality and morbidity^1^ Women often do not meet the intake before pregnancy due to lack of food variety, lack of nutrition information or ignorance^2^. It is believed that limited compliance with iron and folate supplements is a major challenge for the low effectiveness of anaemia-prevention programmes. The World Health Organization (WHO) estimated that 58% of pregnant women in developing countries were anaemic^2^ and later the global prevalence of anaemia for pregnant women was estimated to be 38.2% (95% CI: 33.5–42.6) and for all women of reproductive age was 29.4% (95% CI: 24.5–35.0)^3^. The South African National Health and Nutrition Examination Survey (SANHNES)^4^ estimated anemia prevalence in females of reproductive age to be 23.1% and 41.8% in women, while WHO^5^ earlier estimated 61.3% in women in Africa and 32.5% in women in South East Asia. The latest estimates by WHO^3^ place the prevalence of anaemia in pregnant women in South Africa to be between 20.0 – 39.9% concurring with Shisana et al^4^. One of the programmes of the Department of Health in South Africa is to supply iron and folate supplements to all pregnant women to prevent anaemia as recommended by World Health Organisation^6^. Health workers are expected to supply information about these supplements during counselling to pregnant women^6^. When a pregnant woman visits an antenatal clinic for the first time in South Africa, she is registered and general medical assessment including blood test is done^6^. The Basic Antenatal care plan is then determined and iron and folate supplements are issued. During the second visit signs of anaemia are checked and hemoglobin results are discussed and further nutrition education is given depending on the hemoglobin results. These are supposed to be guidelines to be applied in all antenatal care clinics according to Department of Health^7^.

Casey et al.^8^ established major barriers for effective supplementation programmes as inadequate supply of iron and folate supplements. Additional barriers were found to include inadequate counselling and distribution of iron tablets, difficult access and poor utilization of primary health care services, beliefs against consuming medications during pregnancy and unproven and unscientific fears that taking too much iron may cause too much food or a big baby. Poor diet has also been reported to contribute to multiple micronutrient deficiency during pregnancy^9^. Anaemia impairs human function at all stages and severe anaemia during pregnancy is thought to increase maternal mortality. Anaemia has also been associated with pre-term delivery and low birth weight. Preventive iron supplementation during pregnancy has shown a significant benefit in reducing incidence of anaemia in mothers and low birthweight in neonates^9^. Bopape et al reported a poor dietary intake of iron, folate and vitamin C in pregnant teenagers of Limpopo province in South Africa, which necessitates intervention by health care providers in order to prevent complications that might arise as a result of these dietary inadequacies^10^.

South Africa had in 2002, 8.3% low birth weight infants, with North West Province at 9.1%^7^. It has been reported that South Africa had 21.4% iron deficiency anaemia in pregnant women and North-West Province had 28.6% in 1995^11^; and 27.9% at national and 28.1% in North West Province in 2005^6^ showing little progress over ten years. However, SANHANES One 2013^4^ reported iron deficiency anaemia in pregnant women at national level to be 23.1% and 16.9% in the North West Province. These figures show an improvement particularly in the North West Province over a nine year period. The prevalence however shows the need to continue to provide all pregnant women with folate and iron supplements and nutrition education. The challenge which needs to be addressed is how to encourage the pregnant women to comply with the supplementation regime. In North-West Province primary health care clinics, all pregnant women are given folate and iron supplements to be taken daily^6^,^7^. This study investigated compliance with the consumption of iron and folate supplements by pregnant women in North-West Province, South Africa. Four objectives were formulated as follows:

- To determine the demographics and clinic attendance by pregnant women
- To determine availability of iron and folate supplements at selected primary health care clinics;
- To determine nutrition knowledge of pregnant women and nutrition information the health workers gave with regard to iron and folate supplements to pregnant women;
- To determine consumption of iron and folate supplements and identify factors influencing compliance to the consumption of iron and folate supplements by pregnant women.

## Research methodology

The study design was descriptive, exploratory and cross-sectional. Quantitative techniques were used to obtain information on demographics, attendance and nutrition knowledge of pregnant women and the health workers. The methods were used to obtain in-depth information from the pregnant women on compliance to the consumption of iron and folate supplements. Triangulation was used with in-depth focus group discussion, self - reported questionnaire for pregnant women and a structured interview for the health worker. The study area was Mafikeng local municipality of North West Province in South Africa. The local municipality comprised of 21 clinics at the time of the study, 4 health centres and two hospitals; one of which was a secondary and the other primary health care level. The local municipality had a population of 242 193 people in 2001 according to municipal demarcations12 and 291 478 in 2011^13^. The average number of pregnant women attending antenatal clinics at the 21 clinics and 4 health centers was 1 844 per month^14^. Statistics South Africa^13^ reported 35.7% unemployment rate, 26% matriculation level for people aged 20 and above, and 10% people without schooling in the Mafikeng local municipality.

The sampling design was multi-stage (successive stage of sampling) and non-random using convenience, quota and purposive techniques. Clinics were clustered into four groups as per Mafikeng sub-District clusters, cluster one comprising of one health centre and nine clinics, cluster two with one health centre and seven clinics, cluster three with one health centre and four clinics, and lastly cluster four with one health centre and three clinics^14^. Quotas were used in selecting clinics from each cluster depending on the size of the cluster. Three clinics were selected from clusters one and two while two each were selected from clusters three and four. Health workers and pregnant women were conveniently selected from each clinic. Pregnant women were selected based on the size of each cluster, 19 from cluster one, 16 from cluster two, 10 from cluster three and 12 from cluster four. All pregnant women participated in ten focus groups of between four to seven pregnant women (3 groups each for clusters one and two, two groups each for clusters three and four). The final sample consisted of ten clinics (48% of total), 57 pregnant women, 10 focus groups and 10 health workers (1 each per clinic). The health workers were all professional nurses and midwives and had primary health care training. The health worker on duty on the day of data collection and attending to antenatal care was conveniently selected to participate in the study.

The quantitative data was collected using a standardized self-reported questionnaire on 57 pregnant women. The women had some secondary school education and were competent in basic English. The questionnaire consisted of information on demography, medical history, antenatal care services, and iron and folate nutrition knowledge and education. The pilot study was conducted on six pregnant women from one clinic not included in the final study to determine the feasibility of the study and to test the instruments. Adjustments were made to the methods and instruments after the pilot study. The researcher handed the questionnaire to the participants after explaining the purpose and obtaining their consent. The questionnaire was self completed while he was waiting and available for clarification on questions. Ten focus group discussions were held with between 4 to 7 pregnant women using an interview guide with themes on antenatal services, iron and folate nutrition. The focus groups discussions were conducted on a different date to the self-reporting. The local language, Setswana was used mainly and English was also used depending on the composition of the group. This was done to allow free flow of information by all participants. The researcher, who was a trained registered dietitian and multilingual (South African languages), conducted the focus-group discussions in the same way in each selected clinic, to maintain validity and reliability. The focus group discussion were recorded and later transcribed verbatim and translated to English. The services of the English department at the University of Venda were utilized for the translation of raw data. One professional health worker per clinic was interviewed in English by the researcher using a structured questionnaire consisting of information on antenatal care services rendered same day as the self-reporting by the pregnant women. The interviews with the health worker were conducted at the clinic thus allowing the researcher to make some observations to verify some of the information, e.g. availability of iron and folate supplements. Therefore, all groups and health workers were exposed to similar questions, treatment and behavior by the researcher. Triangulation was used to collect data in order in increase the reliability and accuracy of the results, since it is a strong mixed method that covers data to support a particular hypothesis or theory^15^. The overarching themes for the three groups were iron and folate supplementation and compliance.

Ethical clearance was obtained from University of Venda's higher degrees and ethics committee prior to the collection of data. Furthermore, the permission to conduct the study was granted by the Department of Health in the North-West Province and cooperation was sought from the primary health care clinic managers. The pregnant women were informed about the research and confidentiality matters before agreeing to participate by signing a consent form. The health workers were also requested to give permission by both written consent and oral assent. All participants were given the opportunity to withdraw from the study if they felt the need to do so. The qualitative data was analyzed following the data analysis spiral described by Cresswell^15^. In the spiral, raw data are first organized, perused, classified and synthesized back and forth before final reporting. The quantitative data was analyzed using SPSS version 14. Descriptive statistics such as mean and percentage were used.

## Analysis of results

Twenty eight percent of pregnant women were between the ages 15 to 21 years, 44% between 22 and 30 years and 28% were above 30 years. The majority of pregnant women (62%) were in the third trimester whereas 7% were in the first trimester. Seventy six percent had a life birth history of one to two while 10% had five or more pregnancies. About 12% had suffered between one to four miscarriages with the causes cited as ectopic pregnancy, stress, ammonia, cord knot or unknown. About 88% of pregnant women were unemployed and 44% depended on their mothers for financial support. Most of the pregnant women had some secondary school education and could read and write in English. The racial distribution of the sample was 94.7% black, 3.5% Coloured and 1.8% White. This is comparable to the distribution reported by Stats SA13, which indicated 89.9% black, 2.0% Coloured, 7.8% White, 0.6% Indian and 0.3% other in North West province. About 12% of pregnant women indicated that they had diabetes, hypertension or other disease and were on medication. All ten health workers were professional nurses and midwives with variable experience from three to more than ten years' service.

## Antenatal care clinic attendance

Antenatal care clinic attendance of pregnant women was reported to be high. Both self-reported questionnaire and focus group discussions showed 100% attendance while health workers reported 70% attendance. The difference observed could be due to the fact that health workers were referring to all their clients, whereas the pregnant women were referring to themselves. The frequency of attendance was also depended on trimester of the pregnant women and ranged from once to twice per month and health workers confirmed that most women did honour their appointments. See Table 1 for the responses on frequency of attendance of antenatal care services.

**Table 1:** Antenatal clinic attendance & frequency responses

Responses by method of data collection	Number of participants	Percentage (%)
**Responses from Self-reporting by pregnant women (n = 57)**		
Once a month	37	65
Twice a month	13	23
Four times a month	4	7
More than four times a month	3	5
**Responses from Focus group discussions (n = 10 focus groups of 57 pregnant women)**		
Twice a week	1	1.8
Once per month	13	22.8
Twice per month	4	7.0
4 – 5 Times a month	1	1.8
2–5 Months: 1 per month and then twice	1	1.8
9 month: weekly	1	1.8
First visit	1	1.8
Only came on appointment date	35	61.4
**Responses from Health workers (n = 10)**	
Good	6	60
20 per visit	1	10
Very good	1	10
Supermarket approach	1	10
They come any day, no one is returned	1	10

The pregnant women listed the antenatal care activities that take place on the day of the visit as illustrated in Table 2 below.

**Table 2:** Activities that takes place during antenatal visit

Responses	Number of participants (n = 57)	Percentage (%)
Check child growth and heartbeat	88	1414
Check urine	4	7
Give supplements	4	7
Check discharge	2	3.5
Take blood	2	3.5
Test HIV	2	3.5
Check BP	13	22.8
Check weight	5	8,8
Forget	3	5.3
PMTCT & STI	3	5.3
Told what to bring when giving birth	4	7
Did not respond	3	5.3

PMTCT: Prevention of Mother to Child Transmission STI: Sexually transmitted infections

## Availability of iron and folate supplements

About 95% of the pregnant women said that they were issued with supplements while 100% of health workers said they issued supplements all the time. Nine clinics said that they had stock on the day of data collection and this was confirmed by researcher observation. When asked about actions they take for refill, both pregnant women and health workers said it was a non - issue since supplements were issued in enough quantities and never ran out during the pregnancy period. The responses from health workers on the kinds and procedures followed for supplements issuing is illustrated in Table 3.

**Table 3:** Kinds of supplements issued at a clinic and procedure (n=10; reported verbatim)

Kinds of supplements issued and procedure	Number of health workers (n=10)	Percentage (%)
Folic acid and ferrous sulphate	1	10
First 3 months we give folate and iron, and then iron only, those suspected of HIV, we do not give iron as it is suspected of suppressing bone marrow, we give them vitamin B complex	1	10
Folate, iron and vitamin B complex	1	10
Give according to maternity guidelines	1	10
Folate and vitamin B complex, give iron when you know status (negative) as it is suspected of increasing viral load	1	10
Iron, folate and multivitamin complex	2	20
Iron: 1 per day; Folate: 1 per day; If Hb is low: 2 iron and 1 folate until picked up	1	10
Usually iron, folate for first trimester, and then gluconate and multivitamin complex	1	10
First trimester folate acid: 5 mg 2×/ day and iron: 200mg 1×/ day, and iron thereafter until 6 months post delivery	1	10

The health workers were further asked about the procedure for refills and their responses are illustrated in Table 4.

**Table 4:** Frequency of visits by pregnant women to come for supplements refill

Responses (Verbatim) by Health workers	Number of health workers (n=10)	Percentage (%)
They usually come on scheduled date as it is a monthly supply, some still have them on scheduled date	1	10
They only come once a month	1	10
On monthly basis or when depleted, mostly those on 2 ferrous sulphate per day. Container took 28 tablets	1	10
28 day supply, conditions determine when they will finish, mostly they finish early after 4 months	1	10
When they come to ANC they are given a return date and they come on stipulated date as they are given 28 day supply.	1	10
Every time when they visit	1	10
Normally on stipulated date, but it depend on consumption	1	10
They are usually given return date	2	20
According to maternity guidelines	1	10
They take one month supply, so they come monthly	1	10

ANC: antenatal care

## Nutrition knowledge and education about iron and folate

Pregnant women (68% self-report and 35% focus-group) said they did not know the purpose of consuming iron and folate supplements however, 55.3% gave responses that indicate that they did have knowledge on iron and folate nutrition. Table 5 illustrates the responses by pregnant women on iron and folate nutrition knowledge. Contrary to this, the health workers (60%) said they believed that pregnant women knew the purpose of consuming iron and folate supplements. Furthermore, 60% of the health worker believed that pregnant women knew the consequences of non-compliance whereas 53% of pregnant women said they did not know the purpose of consuming the supplements.

The health workers were not asked how they established their perception that the pregnant women were knowledgeable of the consequences of not consuming iron and folate supplements. About 70% of health workers mentioned low hemoglobin anaemia as a consequence that they had observed in some women.

**Table 5:** The relationship between iron and folate supplements and unborn baby

Responses (verbatim) by pregnant women	Number of participants (n=57)	Percentage (%)
Build child bones, Make child healthy, Strong powerful child, Child grow well, Make child strong in order to live, Assist the child in development, Protect child; I think they give the child nutrients	22	38.5
Boost blood	2	3.5
Prevent miscarriage	1	1.8
Whatever you eat, the baby eat	1	1.8
The child may be blind if not taken	1	1.8
If hypertension, the pill reduce BP to normal	1	1.8
Increase appetite	1	1.8
They make me hungry	1	1.8
I eat a lot after taking them, the child movement may be due to pills	1	1.8
They do something	1	1.8
No relationship	4	7
Do not know	9	15.8

There were conflicting reports between pregnant women and the health workers on whether education on iron and folate nutrition was conducted. Data showed that 81% of pregnant women reported that they were never taught contrary to 100% of health workers who reported that they gave health education including iron and folate information. On further probing, the researcher noted variation in terms of when education was given. About 60% of health workers said they gave health education with every antenatal visit about diet, others said they gave pamphlets, advice to read food labels, or give education every week and during clinic visit. This difference in reporting is expected due to the fact that health workers were likely to report what is expected of them whereas pregnant women reported lived experiences.

## Compliance with the consumption with iron and folate supplements

The data indicates that 93% of pregnant women (self-report and focus group) were consuming iron and folate supplements. All health workers (100%) believed that pregnant women were consuming iron and folate supplements given to them. The few pregnant women who did not consume supplements are those who cited side effects of “make me sick or dizzy”. Health workers were asked about the procedure they follow to measure compliance and their responses were variable as illustrated in table 6.

**Table 6:** Measuring of compliance by health workers

Responses (verbatim)	Number of health workers (n=10)	Percentage (%)
We usually ask if they had finished the treatment	1	10
When they come we check the container, most have finished but others did not finish due to nausea and vomiting. Those who did not finish we educate them	1	10
We repeat Hb test	1	10
Through statistics records	1	10
We interview, the questions are like how is the progress, if they are complying; and if they have problems they usually tell us	1	10
We ask mothers to accompany teenagers and ask the mother about compliance	1	10
We give them 28 day supply, if after 4 weeks they still have supplements we know they are not complying	1	10
You give them health education, if they understand you are sure they are going to comply	1	10
We only hear from them if they complain with side effects, if no complain they are complying	1	10
Usually ask questions and check Hb levels	1	10

The overall summary of the results are illustrated below in Table 7 under different sub-headings with details.

**Table 7:** Summary of the results on compliance to Iron and Folate supplementation

Variables	Self-reported responses	Focus group responses	Health worker responses	Interpretation by Researcher (based on evidence)
Attendance	100%	100%	60%	Very good
Frequency of attendance	65% (once a month) 35% (2× or more per month)	22.8% (once a month) 61.4% (per appointment) 15.8% (more than 1× per month)	Per appointment and 20% said any day	Very good (based on reports, not clinic registers)
Availability of supplements	95%	Not applicable	100%	Very good
Action for refill	Not indicated	No action or procedure in place, collect during visit	Give sufficient until next visit	Refill is probably not important as enough supplements are given until next visit
Knowledge of purpose of supplements by pregnant women	68% (did not know)	35% (did not know)	60% (they know)	No corroboration (probably pregnant women did not know), but Health workers thought the pregnant women knew.
Knowledge of consequences of non-compliance by pregnant women	Not asked	52.6% (did not know or did not respond)	60%	Probably pregnant women had no knowledge
Nutrition education on Iron and Folate	81% (never taught)	Not clear about being taught but 45.6% said they eat food rich in iron and folate	100% (nutrition education on iron and folate)	Pregnant women contradicted themselves; health workers could have been scared to tell the truth. Nutrition education was probably not done Excellent
Compliance	93%	93%	Good (variation in measuring)	

## Discussion

Most of the women were young adults below 30 years with some secondary school education, unemployed and in their third trimester of the pregnancy. They were depended on social grants and financial support from their parents for their livelihood. The health workers were all professional nurses and midwives.

## Antenatal care clinic attendance

Antenatal care clinic attendance of pregnant women was reported to be high in this study. According to Haider, et al^16^ the good attendance of antenatal care clinic such as observed in this study would lead to improved compliance to consumption of iron and folate supplements. In a qualitative study on antenatal attendance conducted in Ghana, Kenya and Malawi, it was reported that an important factor influencing attendance was the supply side, care received and the pregnant women's expectations^17^. Increased attendance of the antenatal clinic in Uganda was also reported^18^ following community and health facility systems strengthening interventions that included counselling. The findings of this study thus are comparable to those reported elsewhere.

## Availability of iron and folate supplements

There was good availability of the tablets in the clinics and thus implying access for the pregnant women of the supplementation pills all the time. Iron and folate supplements were available in all clinics and was provided to all pregnant women. The South African Department of Health protocol stipulates that pregnant women should be given ferrous sulphate of 170 mg and folate of 5mg to be consumed daily^7^. In contrast, WHO recommends a regimen of 60mg iron and 400 µg folate to be taken daily^2^. These differences are of clinical insignificance. The major barrier to effective iron and folate supplementation programmes has been reported by others to be inadequate supply of supplements, whereas pregnant women may benefit from regular micronutrient supplementation^19^.

## Nutrition knowledge and education about iron and folate

Although the pregnant women said that they were not given education on iron and folate nutrition, while 46% said they ate foods which were rich sources of iron and folate. About 55% also indicated to have knowledge about iron and folate nutrition, despite claims of not hav ing been taught. Furthermore, only 12.5% of pregnant women demonstrated understanding of the consequences of not taking iron and folate supplements. It has been suggested^20^ that compliance can be increased by providing women with clear instructions and educating them on the health benefits of tablets. In addition, motivation and awareness can be increased through nutrition education in order to impact on compliance^21^. Provision of supportive and sensitive antenatal care service appears to promote acceptance of service and attendance thus impacting on compliance^22^. Therefore, to reinforce compliance, continuous counseling and education on supplements and balanced diet are important. Dietary diversification is important in the prevention of anaemia and together with iron and folate supplementation programme, it could yield good pregnancy outcome.

## Compliance with the consumption with iron and folate supplements

High compliance was reported in this study. About 80% compliance was also reported in Kenyan pregnant women^23^ supplemented with iron and folic acid, and 69% compliance in Senegalese pregnant women^20^. The above findings are in contrast with the study done in rural areas of Nigeria where non- compliance was reported^24^. Compliance in this study could have been due to good antenatal care clinic visits observed. The attendance of antenatal care clinics may have had an influence on compliance due to the fact that there was frequent contact between the pregnant women and the health workers which could have resulted in regular counseling. Similar findings have been reported^16^, where regular counseling was found to lead to improved consumption of supplements. The frequency of the antenatal care clinic visits could have increased the likelihood of counseling. Furthermore, compliance may be improved by understanding practices regarding pregnancy and provision of maternal services which are culturally acceptable^25^. A study which was done in rural Limpopo^26^ found that pregnant women were complying with consumption of iron and folate supplements as the prevalence of anaemia was lower than earlier reported in 1999. It was also found in the same study that there was a supplementation policy in South Africa which was implemented and it was bearing fruit as compliance with the consumption of iron and folate had improved.

Pregnant women who experienced side-effects were reported to have reduced compliance^27^. In this study, only 3.5% of pregnant women said they were not consuming iron and folate supplements due to side-effects. The side-effects reported by pregnant women were sickness and dizziness, also reported earlier^28^. Weekly supplementation of iron and folic acid in iron deficiency anaemia patients is said to be as good as daily supplementation with added benefits of less adverse reactions and better compliance^29^. Studies among pregnant women in rural Indonesia demonstrated that compliance with the supplementation intake was a serious problem^21^. The two studies in Indonesia revealed 64% (self-reported) and 36% tested in 45 pregnant women in Jakarta and 31% (self-reported) in 107 women in Sulawesi. Pill count is a more accurate method for estimation of adherence to iron and folic acid supplement than self - reported adherence^30^. Age, income, pregnancy spacing, ANC visits, knowledge of folic acid and family encouragement were statistically significant independent positive predictors in their study. Conversely, crowding index, gravidity, and side effects occurrence were statistically significant independent negative predictors.

The findings in this South African study showed a higher compliance than most studies cited here. Compliance is influenced by the taste and quality of tablets as well as side effect experienced. In another study^31^, direct observers were assigned to monitor consumption of oral supplementation tablets by pregnant women. Findings revealed that the deployment of direct observers for monitoring consumption of supplements was feasible and helped to improve compliance. It has been reported that women with high compliance were motivated by the perception of improved health, the insistence by midwives on consumption of supplements and the mention of possible health benefits for the baby^20^. They also reported that those with low compliance mentioned experience of side effects, misunderstanding of continuous consumption throughout the pregnancy and forgetfulness as the reasons. All these studies confirm that regular clinic attendance, counselling and contact with the health worker and quality of supplements influence compliance with the consumption of iron and folate supplements.

## Conclusion

The majority of pregnant women complied with consumption of iron and folate supplements. The compliance was thought to be influenced by the regular good attendance of antenatal care clinic service observed. Availability of iron and folate supplements in clinics was high and most pregnant women received nutrition education on the importance of supplements from the health workers.

## Recommendations

Compliance should be enhanced through nutrition education, monitoring and evaluation of birth outcomes in order to eliminate anaemia related adverse effects.

## Limitations

The study did not follow up the women to determine birth outcomes and correlation with supplementation intake. The study was also limited to Mafikeng Municipality with a homogenous group of pregnant women sharing culture and socio-economic status and can only be generalized to a similar population.
